# Supplementary material for: Specific, sensitive and quantitative protein detection by in-gel fluorescence
Source: Nat Commun. 2023 May 2;14:2505. doi: 10.1038/s41467-023-38147-8 (PMC10154401; doi:10.1038/s41467-023-38147-8)
Supplement: Supplementary file 1 — Supplementary Information [file 41467_2023_38147_MOESM1_ESM.pdf]

Specific, sensitive and quantitative protein detection by in-gel fluorescence

Supplementary Information

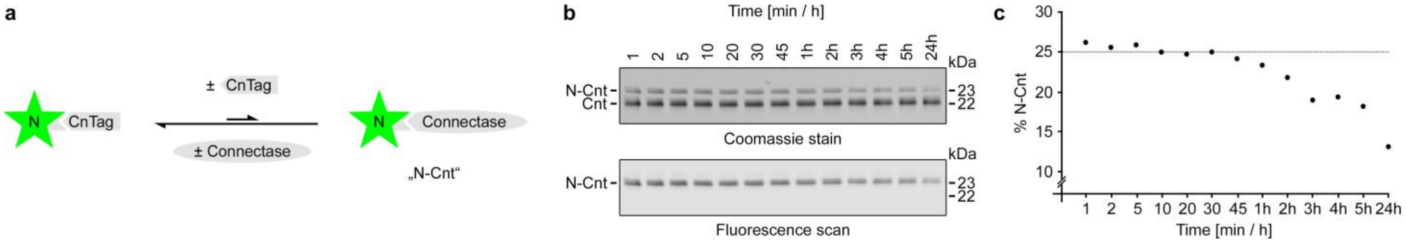

Supplementary Figure 1: N-Cnt formation. Connectase forms an amide-bonded reagent, N-Cnt, with substrates bearing a recognition sequence (A). To visualize this process, equimolar quantities (5 µM) of Connectase and a fluorescent peptide substrate were mixed. The reaction was stopped at the indicated time points and analyzed via SDS-PAGE (B). The Coomassie-stained gel shows both unmodified Connectase (Cnt) and fluorophore-conjugated N-Cnt. A fluorescence scan of the same gel (before Coomassie staining) shows only fluorophore-conjugated N-Cnt. A densitometric quantification (C) shows that roughly 25% N-Cnt is formed and that the equilibrium is already reached after 1 min. The mixture is stable for about 1h at room temperature. Note that the X-axis shows the same (non-linear) incubation times as the gel (B). Source data are provided as a Source Data file.

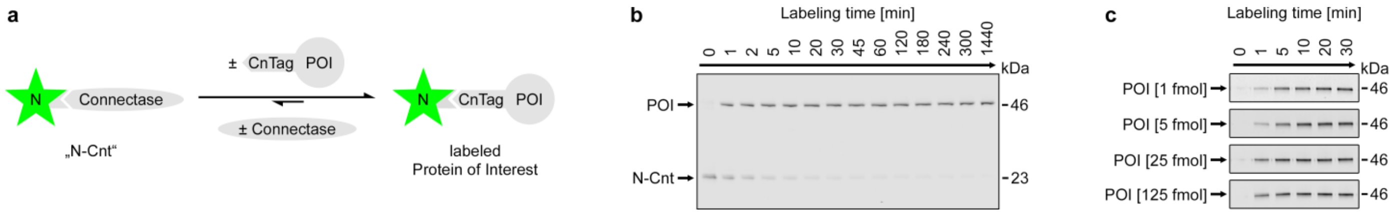

Supplementary Figure 2: Protein labeling.

(A) Fluorophore-conjugated Connectase (N-Cnt) can transfer its fluorophore to a CnTagged protein of interest.

(B) To visualize this process, ~6 fmol N-Cnt were mixed with 125 fmol POI (Ubiquitin-activating enzyme E1 (46 kDa)), incubated for up to 24h, and analyzed via in-gel fluorescence. Sharp and intense POI bands are observed after a few minutes of incubation. The N-Cnt band disappears after ~20 minutes, indicating an almost complete fluorophore transfer. Source data are provided as a Source Data file.

(C) To study labeling rates at different POI concentrations, a similar experiment was conducted with samples containing 0.2 - 25 nM POI (corresponding to 1 - 125 fmol on the gel). The different POI quantities resulted in bands of different intensities, as shown in Figure 4. To compare the labeling rates, we adjusted the brightness/contrast settings, so that all 30 min - bands appear equally intense. In this representation, it can be seen that the labeling reaction is faster for more concentrated samples and that the maximum signal is obtained after 30 min in all cases. Source data are provided as a Source Data file.

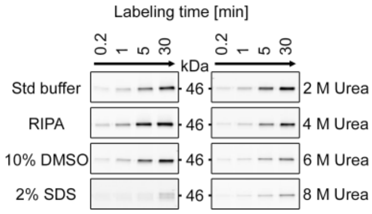

Supplementary Figure 3: Protein labeling rates in different buffers. A CnTagged POI (Ubiquitin-activating enzyme E1 (46 kDa)) was labeled in different buffers. The reaction was stopped at the indicated time points and analyzed via in-gel fluorescence. The experiment was conducted with very low POI concentrations (0.2 nM, corresponding to 1 fmol on the gel), which result in lower labeling rates (Figure S2C). At higher POI concentrations, the differences between the buffers would be less visible. Approximate relative labeling rates on the gels are: RIPA (2x) > 2M urea (1.2x) > Std buffer = 10% DMSO (1x) > 4 M urea (0.9x) > 6 M urea (0.2x) > 8 M urea (0.1x). Note, however, that the use of detergents and chaotropes may result in inhomogeneous samples (different POI conformations, partially detergent-bound or partially unfolded) and should therefore be avoided for quantifications (where possible). Std buffer is composed of 50 mM Acetate, 50 mM MES, 50 mM HEPES, 50 mM KCl, 150 mM NaCl, pH 7.0. Source data are provided as a Source Data file.

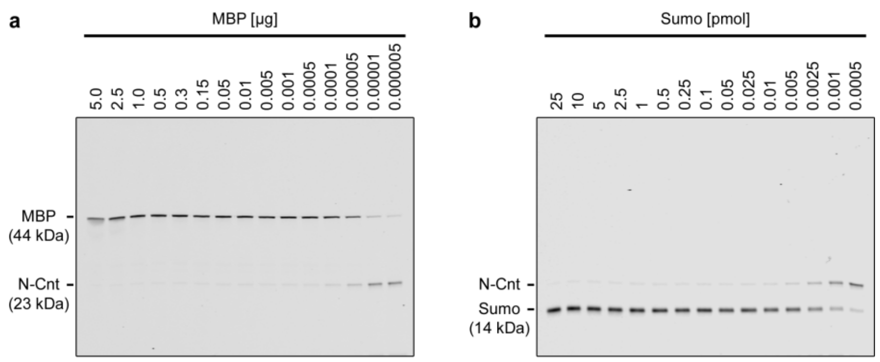

Supplementary Figure 4: Replication of published sensitivity tests for other in-gel detection methods.

(A) Different amounts of CnTagged MBP (Maltose-Binding Protein) in *E. coli* cell lysate (2.5 µg total protein) were detected via Connectase-mediated in-gel fluorescence. Lanes 1-8 correspond to a similar experiment used to determine the detection limit of His_{12} tags with super-chelator probes (0.01 µg or 0.2 pmol[1]). Source data are provided as a Source Data file.

(B) Different amounts of CnTagged Sumo (Small ubiquitin-like modifier) were detected via Connectase-mediated in-gel fluorescence. Lanes 1-8 correspond to a similar experiment used to determine the detection limit of His_6 tags with super-chelator probes (0.1 pmol[2]). Both experiments (A, B) are in accordance with the results shown in Figure 4 (detection limit ~0.1 fmol). Source data are provided as a Source Data file.

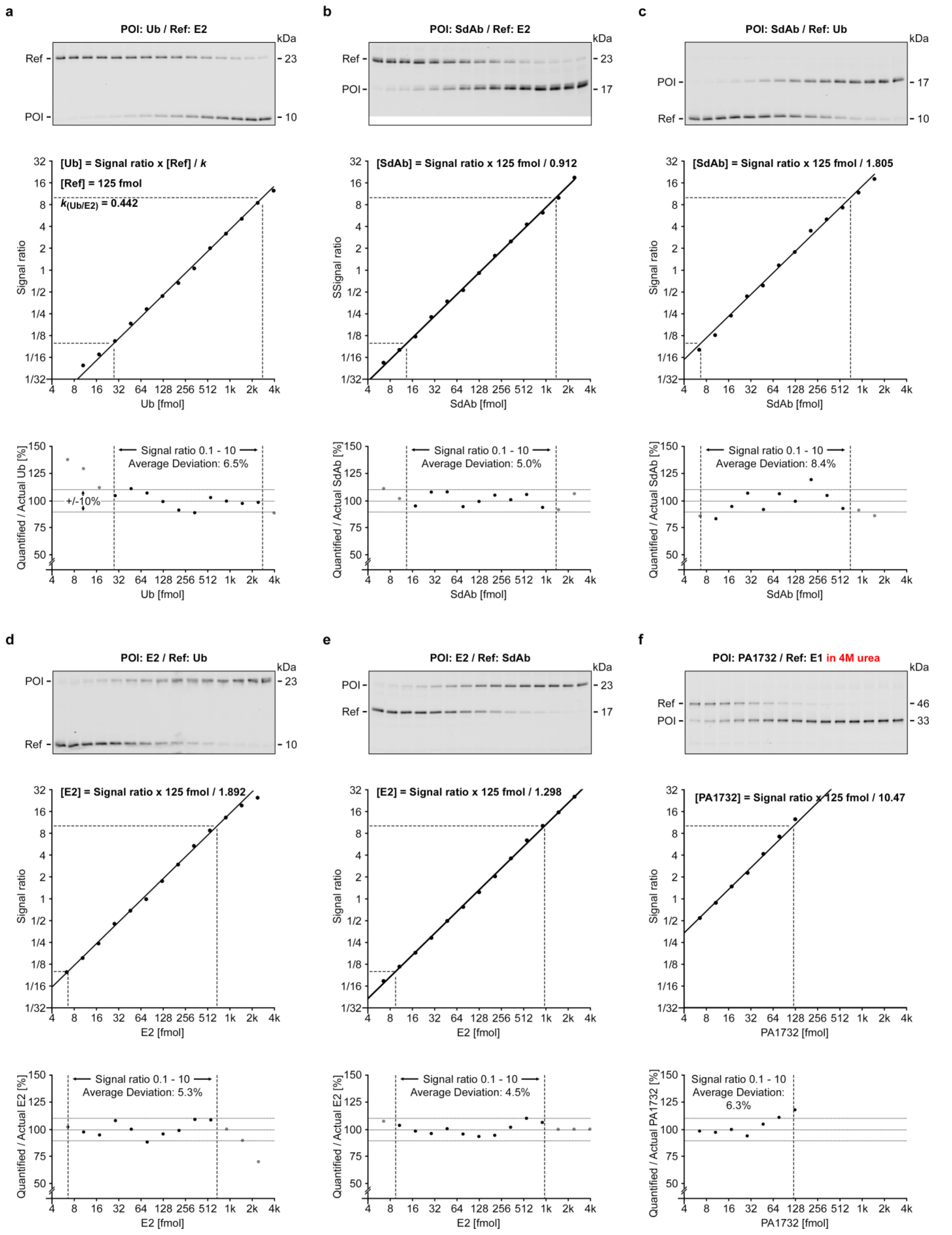

Supplementary Figure 5 (related to Figure 6): Protein quantification with the in-gel fluorescence competition assay.

(A-E) Shown are five competition experiments with constant amounts (125 fmol) of a reference protein and increasing quantities of a protein of interest. The POI/reference pairs are Ubiquitin (Ub) / Ubiquitin-conjugating enzyme E2 (A), Single-domain Antibody (SdAb) / E2 (B), SdAb / Ub (C), E2 / Ub (D) and E2 / SdAb (E). The reactions were separated on polyacrylamide gels (top layer), followed by the densitometric quantification

of fluorescent protein bands. The resulting data is represented in plots (middle layer), revealing a linear relationship between signal ratio and protein of interest quantities in all cases. The individual curves are shifted in Y-direction by the factor *k* (see Eq.2; $k_{(Ub/E2)}$ = 0.442 (A); $k_{(SdAb/E2)}$ = 0.967 (B); $k_{(SdAb/Ub)}$ = 1.805 (C); $k_{(E2/Ub)}$ = 1.892 (D); $k_{(E2/SdAb)}$ = 1.021 (E)). The linear relationship allows the quantification of target proteins in unknown samples (see Figure 7). The error of such quantifications is represented in plots (bottom layer). The most accurate values are obtained at signal ratio 0.1 – 10 (dotted lines). Note that the experiments were conducted with similar, but not entirely identical parameters (e.g. different protein preparations). The SDS-gels show different POI quantities (e.g., 3.953 – 3953 fmol for Ub/E2 and 1.474 - 1474 fmol for SdAb/Ub). Source data are provided as a Source Data file.

(F) A similar competition experiment for the quantification of PA1732 from *P. aeruginosa*, a protein that aggregates in inclusion bodies when expressed in *E. coli* [3]. For the experiment, PA1732 inclusion bodies were unfolded in 8 M urea and mixed with 0.5 vol. reference protein and 0.5 vol. labeling reagent for a final urea concentration of 4 M. Source data are provided as a Source Data file.

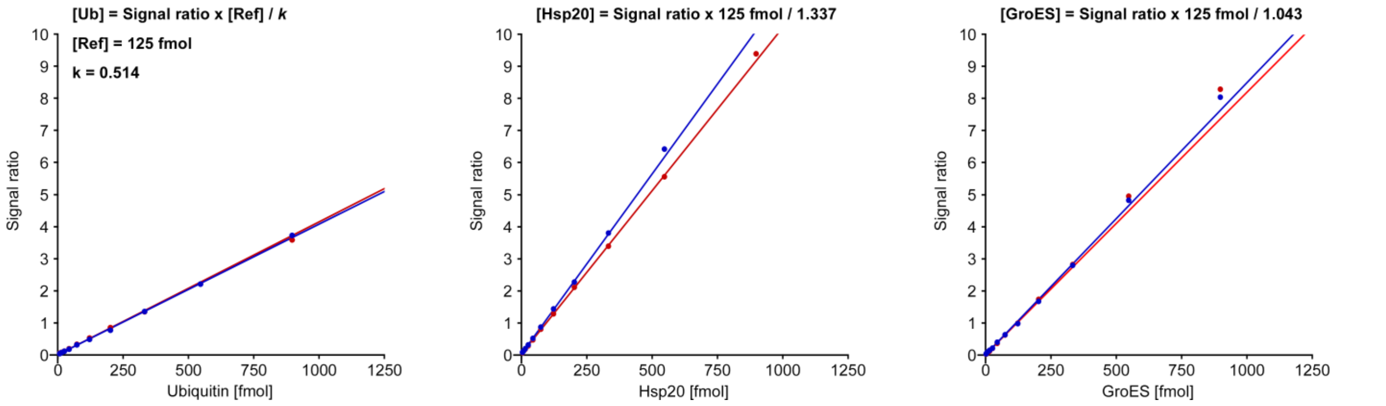

Supplementary Figure 6: The plots in Figure 6 (see main text) on a linear scale. The protein-pair specific constant *k* is seen as a Y-shift on the log-scale (Figure 6) and as a different slope on the linear scale (this figure). Shown is a smaller signal ratio range compared to Figure 6 (i.e., up to signal ratio 10).

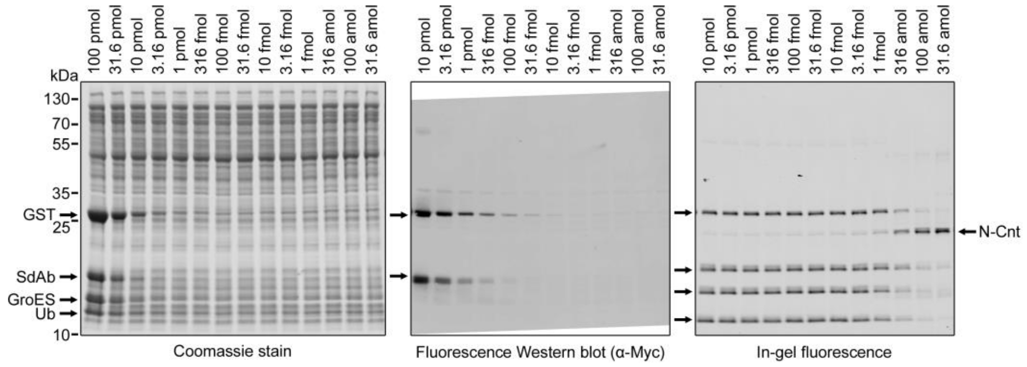

Supplementary Figure 7: Protein detection by Coomassie staining (left), Western blot (middle), or in-gel fluorescence (right). Four proteins, cMyc- or CnTagged Glutathione-S-Transferase (GST), Single-domain Antibody (SdAb), GroES and Ubiquitin (Ub), were serially diluted in *E. coli* cell extract, separated via SDS-PAGE and visualized. In the Western blot, GST and SdAb were detected with good sensitivity, while GroES and Ub gave only a weak signal. The labeling reactions for in-gel fluorescence were conducted before mixing the proteins (i.e., no competition reaction; see methods). Source data are provided as a Source Data file.

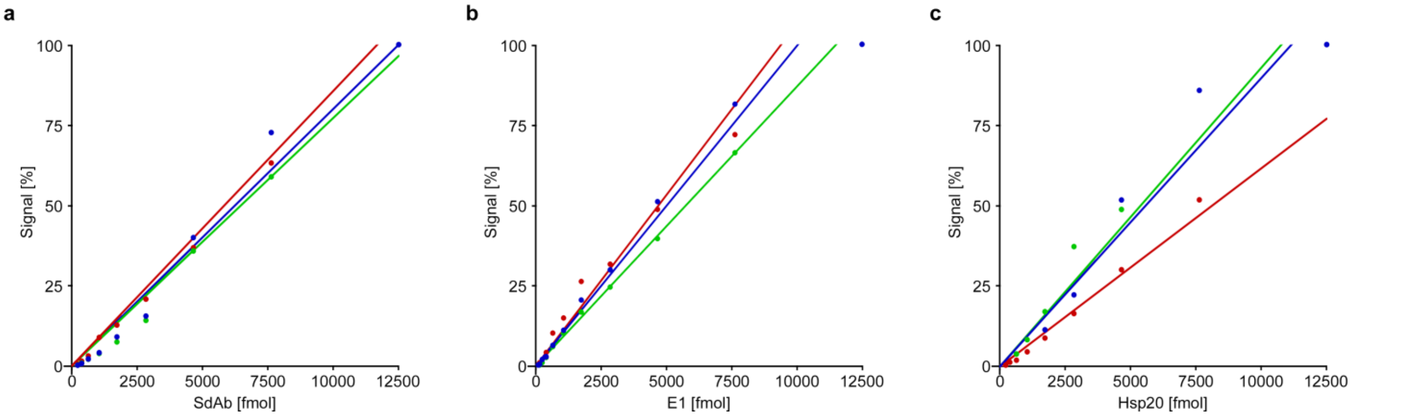

Supplementary Figure 8: The plots in Figure 8 (see main text) on a linear scale. Compared to log-log charts (Figure 8), this representation conceals deviations between quantification data and the data fit at lower POI quantities. The data point at 12500 is 100% in all experiments (normalized data, see main text).

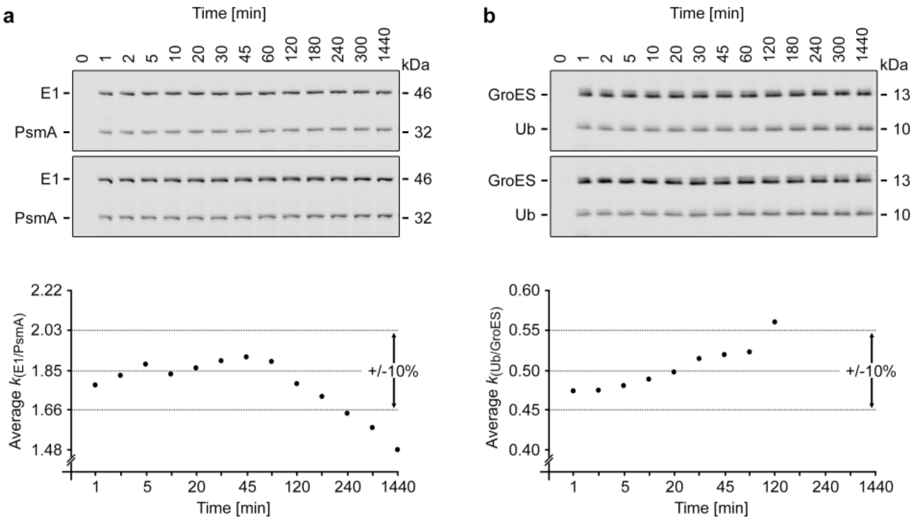

Supplementary Figure 9: In-gel fluorescence competition assays are time sensitive. Equal quantities of two CnTagged protein pairs, Ubiquitin-activating enzyme E1 and proteasome subunit alpha (PsmA, A), or Ubiquitin (Ub) and GroES (B), were mixed and labeled with fluorophore. The labeling reaction was stopped at the indicated time points. The samples were then visualized via in-gel fluorescence, following densitometric analysis of the signal ratios (plots). The obtained signal ratios (expressed as $k_{(POI/Ref)}$ (see Eq. 3)) remain constant over short time periods, but change over longer time periods. Consequently, the same labeling time (we recommend 30 min) should be used for all quantitative in-gel fluorescence assays to ensure comparability. Source data are provided as a Source Data file.

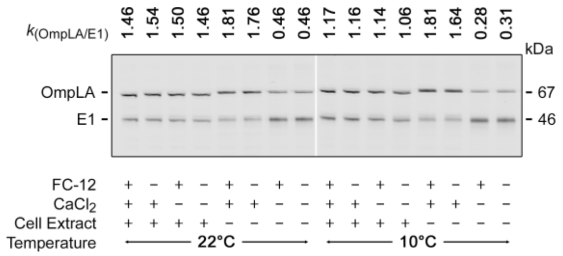

Supplementary Figure 10: In-gel fluorescence competition assays are sensitive to the labeling buffer and the labeling temperature. Equal quantities of CnTagged Outer membrane phospholipase A1 (OmpLA) and ubiquitin-activating enzyme (E1) were mixed and labeled in different reaction buffers and at different temperatures. The reactions were visualized via in-gel fluorescence and the signal ratios were determined densitometrically (expressed as $k_{(OmpLA/E1)}$ (see Eq. 3); shown are the average values of two independent experiments). The results show that the OmpLA band is more intense, and the signal ratio therefore higher, when the labeling reaction is performed at 22°C. Furthermore, it is more intense when the labeling reaction was performed in presence of the detergent FC-12. This effect was, however, only seen in absence of cell

extract. One possible explanation for this is that both the membrane fragments in the extract and the detergent are sufficient to keep the membrane protein OmpLA in solution. $CaCl_2$, which has been shown to bind to OmpLA, had a minor effect on the signal ratios. Overall, we conclude that labeling buffer and temperature matter and therefore should be kept constant in comparative experiments. Source data are provided as a Source Data file.

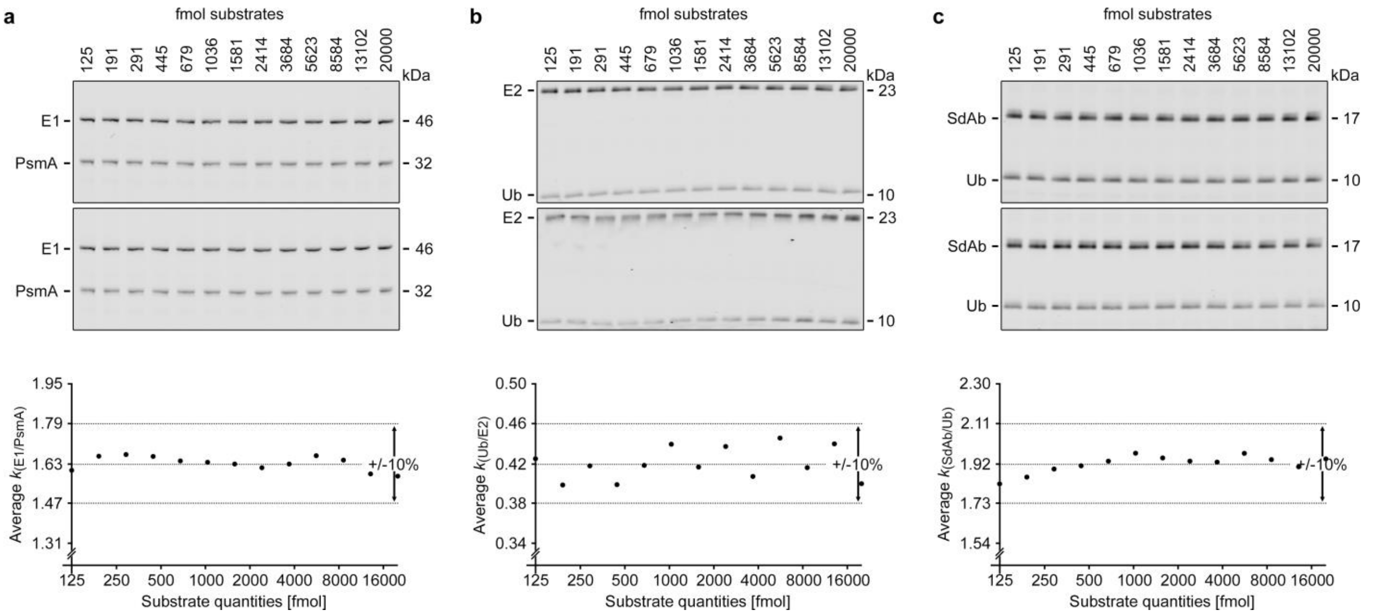

Supplementary Figure 11: *k* values remain constant over a wide range of concentrations. Equimolar quantities of two CnTagged proteins, Ubiquitin-activating enzyme E1 and Proteasome subunit Alpha (PsmA, A), Ubiquitin (Ub) and Ubiquitin-conjugating enzyme E2 (B), and Single-domain Antibody (SdAb) and Ubiquitin (C), were mixed and labeled with Connectase. The reactions, which contained a total CnTagged protein concentration of 35 nM - 5333 nM, were analyzed via in-gel fluorescence. The resulting band intensities were analyzed densitometrically to determine signal ratios (expressed as $k_{(POI/Ref)}$ (see Eq. 3) in the plots). The maximum protein quantity shown in the gels corresponds to 0.5 µg (10 pmol E1). Source data are provided as a Source Data file.

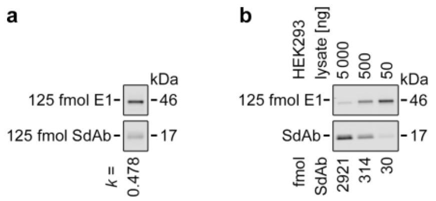

Supplementary Figure 12: Quantification of SdAb protein expression in HEK293 cells (related to Figure 5A).

(A) The *k* value for a SdAb / E1 pair was determined by measuring the signal ratio in a 1:1 mixture (see Eq. 3). The accuracy of this value can be increased by measuring replicates and different substrate ratios (Figure 6). Source data are provided as a Source Data file.

(B) HEK293 cells expressing SdAb (shown in Figure 5A) were lysed and mixed with E1 protein as a reference. The resulting signal ratios (11.15, 1.198 and 0.114) and the determined *k* value (A) can be used to quantify the number of SdAb molecules in each sample with [SdAb] = 125 fmol x Signal ratio / 0.478 (see Eq. 2). The results, 2921 fmol (5000 ng sample), 314 fmol (500 ng sample) and 30 fmol (50 ng sample), can be averaged to conclude that each µg cell lysate contains 603 fmol SdAb. Source data are provided as a Source Data file.

Supplementary Note 1 - Assay Protocol

For quantitative analysis: sample preparation

(1) Prepare samples containing CnTagged protein of interest. Include controls to verify the linearity of the assay. Important: All samples should contain the same buffer (or the same quantities of cell extract), the same volume and should be prepared in the same tubes.
(2) Mix 20 µl of each sample to be analyzed with 20 µl reference protein solution (100 nM).
(3) Optional: For absolute quantification, mix 20 µl of a sample of known concentration (100 nM) with 20 µl reference protein solution (100 nM). This sample is used to determine k (see below). Use replicates to increase the accuracy and/or a dilution series to verify the linearity of the assay.

Labeling and SDS-PAGE analysis

(1) Mix 5 µl of a suitable (see below) fluorescent peptide (10 µM) with 5 µl of *M. mazei* Connectase (10 µM) in detergent-free buffer (e.g., PBS or buffer A (see methods)).
(2) Incubate for 1 min at room temperature (do not store ≥60 min (Supplementary Figure 1)).
(3) Add 2.49 ml buffer to obtain a 20 nM labeling solution.
(4) Mix 20 µl labeling solution with 40 µl sample (see above: sample preparation; consider a control lane without CnTagged POI).
(5) Incubate for ≥5 min (qualitative analysis) or 30 min (quantitative analysis) at room temperature.
(6) Transfer 45 µl of the reaction to a fresh tube containing 15 µl 4x SDS-PAGE loading buffer (optional: heat incubation).
(7) Load 5 µl on an SDS-gel. The rest of the sample can be stored in the freezer and used for future gels.
(8) Run the gel.
(9) Image on a suitable fluorescence imager.
(10) Optional: Store the gel in fixation solution (50% methanol / 10% acetate).

For quantitative analysis: densitometric analysis

(1) Obtain band density values with the instrument software or with the free program ImageJ.
(2) In Excel, divide all protein of interest signals by the reference protein signals to obtain the signal ratio.
(3) For relative quantifications, compare the signal ratio of the different samples. A twice as high signal ratio (e.g., 1.5 vs 0.75) signifies twice as much protein of interest.
(4) For absolute quantifications, determine the signal ratio for the sample of known concentration (100 nM). This value is identical with *k* (see main text, Eq. 3).
(5) Use the relationship $[\mathrm{POI}] = \frac{\text{Signal ratio x [Ref]}}{k}$ to determine absolute target protein quantities in each sample.

Supplementary Note 2 - Notes

Adapting the protocol. In the protocol, equal volumes of reference protein, sample, reagent and SDS loading dye are mixed (i.e., 1:1:1:1) and 5 µl of this mixture are applied to the SDS-gel. The reference protein solution is at 100 nM and 1.25 µl (i.e., 125 fmol) end up in each lane. The labeling reagent contains 20 nM fluorescent peptide and 20 nM Connectase, leading to the formation of ~ 5 nM N-Cnt (see Figure S1), of which 1.25 µl (i.e., 6 fmol) end up in each lane. These numbers can be adapted, if necessary.

Non-linear signal ratio-to-substrate relationships. Quantitative assays require some practice to make sure that all samples are prepared in the same way (buffer, volume, tube) and that the labeling reactions are performed in the same way (time, temperature). If a non-linear signal ratio-to-substrate relationships persists, the POI conformation or assembly state may change in a concentration-dependent manner or the sample may contain different POI species (e.g. different folding states). To avoid such effects, the sample preparation may be optimized and detergent- / chaotrope-free buffers should be used. If the problem persists, quantifications require a standard curve.

Reaction buffer. *M. mazei* Connectase is active over a wide range of pH values and salt concentrations. It tolerates relatively high levels of detergents, DMSO and urea (Supplementary Figure 3). Thus, it is usually not necessary to adapt the sample buffer for qualitative analysis. For quantitative analyses, the detergent-free buffer should be used to avoid sample inhomogeneities (see above) and dilutions should be made with the same buffer. For example, if the POI is in cell extract, dilutions should be made with POI-free cell extract. The labeling reagent (see above) should be prepared in detergent-free buffer without impurities (e.g., BSA).

Avoiding sample loss. For quantitative analyses, it is important to avoid sample loss. We recommend the use of PCR tubes or low protein binding tubes. For dilution series, *E. coli* cell extract proved effective for avoiding sample loss.

Residual fluorescent peptide. In high percentage gels, residual fluorescent peptide can be detected as an extra band slightly above the level of the bromophenol blue dye front. This can be avoided by running the gels slightly longer. In presence of cell extract, this band may disappear in the course of the labeling reaction, possibly because of peptide-degrading proteases.

N-Cnt band. The extent to which the N-Cnt band remains visible depends on the ratio between CnTagged substrates and N-Cnt (see Figure 1). In presence of >25 fmol CnTagged POI (Figure 4), it should be faint compared to the POI bands. If an unexpectedly strong N-Cnt band is observed, this indicates protein adsorption on the tube. This can be avoided by transferring the reaction to a fresh tube containing SDS loading buffer. In addition, longer incubation times or lower N-Cnt quantities may be tested.

Reaction temperature. We performed all assays at room temperature (literally). In principle, *M. mazei* Connectase is most active at 50°C, but we found that its ~7.7x decreased reactivity at room temperature[4] is still more than sufficient to perform the labeling reaction in a few minutes. If necessary, the reaction temperature can be decreased to 10°C (Supplementary Figure 10). For comparative quantifications, however, it is important to label all samples at the same temperature.

Fluorophore-induced band shift. The labeling with fluorophore increases the size of the target protein by ~2 kDa and therefore results in a small band shift.

The protein of interest. We typically clone the CnTag sequence, PGAFDADPLVVEI (MtrA residues 155-167), plus a 5 amino acid linker after the start-methionine of the protein of interest. This start-methionine is removed completely during expression in every protein investigated so far, so that the CnTag is exposed automatically. The choice of the 5 amino acid linker affects the reactivity and stability of the CnTagged POI. In our experience, AAAGA (recommended) or GGGGG are "safe" choices, which work reasonably well. Acidic linkers, such as SEEGE (MtrA residues 168-172) or DDDDD led to higher labeling rates, but also made some proteins susceptible to partial proteolytic degradation when

produced *in E. coli*. For example, the SEEGE linker led to 10% - 50% cleavage of the CnTag for roughly 1/3 of all tested proteins (2/3 were not affected).

Comparability of signals on different gels. In contrast to Western blots, technical replicates of quantitative in-gel fluorescence competition assays produce almost identical signal ratio data (not raw signals). Therefore, signal ratios on different gels may be compared if all assay parameters are kept identical. Nevertheless, it is good practice to include controls on each gel. For example, the same sample could be included in two series of experiments (i.e. labeling reactions) that should be compared. Note that signal ratios cannot be compared between different preparations of the same protein or between new and old protein preparations. In these cases, it must first be validated that the samples behave the same way.

Reference protein. Any well behaved homogeneous CnTagged protein can be used as a reference protein, if its SDS-PAGE migration characteristics differ from the target proteins and N-Cnt (~24 kDa). For our assays, we often used camelid single-domain antibodies (17 kDa).

Fluorescent peptide reagent. We have used Cy5.5-RELASKDPGAFDADPLVVEI as a reagent. The type of fluorophore can be varied depending on the instrument for detection. The use of (Infra-)red fluorophores is expected to result in a better signal-to-noise ratio. The peptide sequence is based on the *M. mazei* Connectase (entry mma:MM_2909 in the KEGG database) interaction partner MtrA (MM_1543, residues 148 - 167). If a Connectase variant from a different organism is used, it should be adapted to the respective MtrA sequence of that organism.

Connectase from different organisms. We have chosen *M. mazei* Connectase, because it expresses well, is reasonably stable and soluble at high concentrations, and has an activity optimum at neutral pH and moderate salt concentrations. It is likely, however, that more favorable Connectase homologs exist among the highly diverse range of sequences. These homologs may recognize mutually exclusive recognition sequences, allowing two separate labeling reactions with different fluorophores in parallel (multiplexing).

Stability of the N-Cnt labeling reagent. The labeling reagent is stable for at least 1h at room temperature, but starts to precipitate slowly afterwards.

Storing the SDS-gel. The gel should be stored in fixation solution (50% methanol / 10% acetate). The Cy5.5 fluorophore employed in this paper is not particularly light sensitive. However, for longer storage (several days), the gel box should be wrapped in aluminium foil and stored at low temperatures. The signal is then stable for weeks, although small proteins tend to diffuse.

Sharp bands. Quantitative analyses are more accurate with sharper gel bands. We therefore recommend the use of commercial polyacrylamide gels and the application of low and identical volumes (e.g., 5 µl) of sample.

Supplementary References

1 Bruchert, S., Joest, E. F., Gatterdam, K. & Tampe, R. Ultrafast in-gel detection by fluorescent super-chelator probes with HisQuick-PAGE. *Commun Biol* **3**, 138, doi:10.1038/s42003-020-0852-1 (2020).

2 Raducanu, V. S., Isaioglou, I., Raducanu, D. V., Merzaban, J. S. & Hamdan, S. M. Simplified detection of polyhistidine-tagged proteins in gels and membranes using a UV-excitable dye and a multiple chelator head pair. *J Biol Chem* **295**, 12214-12223, doi:10.1074/jbc.RA120.014132 (2020).

3 Fuchs, A. C. D. *et al.* The Architecture of the Anbu Complex Reflects an Evolutionary Intermediate at the Origin of the Proteasome System. *Structure* **25**, 834-845 e835, doi:10.1016/j.str.2017.04.005 (2017).

4 Fuchs, A. C. D. *et al.* Archaeal Connectase is a specific and efficient protein ligase related to proteasome β subunits. *Proceedings of the National Academy of Sciences* **118**, e2017871118, doi:10.1073/pnas.2017871118 (2021).
