# Supplementary material for: Specific, sensitive and quantitative protein detection by in-gel fluorescence
Source: Nat Commun. 2023 May 2;14:2505. doi: 10.1038/s41467-023-38147-8 (PMC10154401; doi:10.1038/s41467-023-38147-8)
Supplement: Supplementary file 3 — Description of Additional Supplementary Files Document [file 41467_2023_38147_MOESM3_ESM.pdf]

Description of Additional Supplementary Files Document

Supplementary Dataset 1 - Sequences of proteins used in the study.
