# Supplementary material for: Specific, sensitive and quantitative protein detection by in-gel fluorescence
Source: Nat Commun. 2023 May 2;14:2505. doi: 10.1038/s41467-023-38147-8 (PMC10154401; doi:10.1038/s41467-023-38147-8)
Supplement: Supplementary file 5 — Reporting Summary [file 41467_2023_38147_MOESM5_ESM.pdf]

nature portfolio

Corresponding author(s): Adrian Fuchs

Last updated by author(s): Apr 4, 2023

Reporting Summary

Nature Portfolio wishes to improve the reproducibility of the work that we publish. This form provides structure for consistency and transparency in reporting. For further information on Nature Portfolio policies, see our Editorial Policies and the Editorial Policy Checklist.

Statistics

For all statistical analyses, confirm that the following items are present in the figure legend, table legend, main text, or Methods section.

n/a	Confirmed	
☐	☒	The exact sample size (*n*) for each experimental group/condition, given as a discrete number and unit of measurement
☐	☒	A statement on whether measurements were taken from distinct samples or whether the same sample was measured repeatedly
☒	☐	The statistical test(s) used AND whether they are one- or two-sided<br>*Only common tests should be described solely by name; describe more complex techniques in the Methods section.*
☒	☐	A description of all covariates tested
☒	☐	A description of any assumptions or corrections, such as tests of normality and adjustment for multiple comparisons
☒	☐	A full description of the statistical parameters including central tendency (e.g. means) or other basic estimates (e.g. regression coefficient) AND variation (e.g. standard deviation) or associated estimates of uncertainty (e.g. confidence intervals)
☒	☐	For null hypothesis testing, the test statistic (e.g. *F*, *t*, *r*) with confidence intervals, effect sizes, degrees of freedom and *P* value noted<br>*Give P values as exact values whenever suitable.*
☒	☐	For Bayesian analysis, information on the choice of priors and Markov chain Monte Carlo settings
☒	☐	For hierarchical and complex designs, identification of the appropriate level for tests and full reporting of outcomes
☒	☐	Estimates of effect sizes (e.g. Cohen's *d*, Pearson's *r*), indicating how they were calculated

Our web collection on statistics for biologists contains articles on many of the points above.

Software and code

Policy information about availability of computer code

Data collection	Image Studio 5.2.5; Unicorn 5.1.0
Data analysis	ImageJ 1.52a with Bio-Formats 6.11.0 plugin; Colab AlphaFold implementation with Jackhmmer (https://colab.research.google.com/github/sokrypton/ColabFold/blob/main/beta/AlphaFold_wJackhmmer.ipynb#scrollTo=pc5-mbsX9PZC)

For manuscripts utilizing custom algorithms or software that are central to the research but not yet described in published literature, software must be made available to editors and reviewers. We strongly encourage code deposition in a community repository (e.g. GitHub). See the Nature Portfolio guidelines for submitting code & software for further information.

Data

Policy information about availability of data

All manuscripts must include a data availability statement. This statement should provide the following information, where applicable:

- Accession codes, unique identifiers, or web links for publicly available datasets
- A description of any restrictions on data availability
- For clinical datasets or third party data, please ensure that the statement adheres to our policy

Source data are provided with this paper. The primary data have also been deposited in the Mendeley public repository (https://data.mendeley.com/datasets/3n47z3g33b). The crystal structure data used in this study are available in the PDB database under accession code 6ZW0 (https://www.rcsb.org/structure/6ZW0).

Human research participants

Policy information about studies involving human research participants and Sex and Gender in Research.

Reporting on sex and gender	N/A
Population characteristics	N/A
Recruitment	N/A
Ethics oversight	N/A

Note that full information on the approval of the study protocol must also be provided in the manuscript.

Field-specific reporting

Please select the one below that is the best fit for your research. If you are not sure, read the appropriate sections before making your selection.

☒ Life sciences ☐ Behavioural & social sciences ☐ Ecological, evolutionary & environmental sciences

For a reference copy of the document with all sections, see nature.com/documents/nr-reporting-summary-flat.pdf

Life sciences study design

All studies must disclose on these points even when the disclosure is negative.

Sample size	No sample size calculation was performed. The number of detected proteins (10 proteins in Figure 3A; 3 proteins in Figure 4, 5A, 5B, 6 and 8) was chosen to support the concepts presented in the article.
Data exclusions	No data were excluded.
Replication	Three independent experiments were conducted for the preparation of Figures 4 and 8. Two independent experiments were conducted for the preparation of Figures 6, S9 and S11. In addition, the findings in Figure 6 were supported by a set of similar experiments (Figure S5). All attempts at replication were successful. The experiments shown in Figures 3, 5, S1-S5, S7, S10 and S12 were only performed once in this exact way, as they are in line with similar experiments and results generated throughout our studies.
Randomization	Not relevant to the study. Samples were not allocated into different experimental groups
Blinding	Blinding was not possible as gel/blot images were analysed. Such images cannot be analysed without seeing them, so blinding was not possible.

Reporting for specific materials, systems and methods

We require information from authors about some types of materials, experimental systems and methods used in many studies. Here, indicate whether each material, system or method listed is relevant to your study. If you are not sure if a list item applies to your research, read the appropriate section before selecting a response.

Materials & experimental systems

n/a	Involved in the study	
☐	☒	Antibodies
☐	☒	Eukaryotic cell lines
☒	☐	Palaeontology and archaeology
☒	☐	Animals and other organisms
☒	☐	Clinical data
☒	☐	Dual use research of concern

Methods

n/a	Involved in the study	
☒	☐	ChIP-seq
☒	☐	Flow cytometry
☒	☐	MRI-based neuroimaging

Antibodies

Antibodies used	Monoclonal mouse anti cMyc 9E10 antibody (Thermo, #13-2500, Lot VK307345)<br>IRDye 680RD goat anti-mouse antibody (Licor, Lot #D10512-15; 926-68070)

Validation	Primary antibody validation available under https://www.thermofisher.com/antibody/product/c-Myc-Antibody-clone-9E10-Monoclonal/13-2500

Eukaryotic cell lines

Policy information about cell lines and Sex and Gender in Research

Cell line source(s)	HEK293 cells (DMSZ no.: ACC 305) and Spodoptera frugiperda Sf9 cells (DSMZ no.: ACC 125; gifts from Dr. Birte Hernandez Alvarez)
Authentication	Cells were not authenticated.
Mycoplasma contamination	Cells were not tested for mycoplasma contamination.
Commonly misidentified lines (See ICLAC register)	No commonly misidentified cell lines were used.
