# Supplementary material for: Specific, sensitive and quantitative protein detection by in-gel fluorescence
Source: Nat Commun. 2023 May 2;14:2505. doi: 10.1038/s41467-023-38147-8 (PMC10154401; doi:10.1038/s41467-023-38147-8)
Supplement: Supplementary file 6 — Source Data [file 41467_2023_38147_MOESM6_ESM.zip › Source Data/Reagent analysis/31334121_U2441FG300_3_COA_U2441FG300-3-PE7161.pdf]

CERTIFICATE OF ANALYSIS

Product Name	peptide Cy5.5
Order ID	U2441FG300_3
Lot No.	U2441FG300-3/PE7161
Sequence	RELASKDPGAFDADPLVVEI
Modification	N-T:Cy5.5,
Length	20AA
Storage	-20°C
Recommended Solvent*	dimethyl sulfoxide(Analytical grade)
comments	TFA salt

Test Items	Specifications	Results
Molecular Weight	Theoretical MW: 2707.16	Consistent
HPLC purity	≥95.0%	95.6%
Appearance	Bluelyophilizedpowder	Conforms
Gross Weight	9 mg	10*0.9mg

*Note: Above recommended solvents for reference only. If there is any request for detailed dissolution conditions,we suggest you choose our 'Peptide Solubility Test Service'.

Caution:
For laboratory or further manufacturing use only. Not intended for household use. If you have any questions about the Certificate of Analysis, please contact our customer service representative at 1-877-436-7274 (Toll-Free), or 1-732-885-9188.

Certified by: Gary Chen Date:10-18-2020
Peptide Production Director

Thank you for your patronage to our Peptide services! To maintain this working relationship, we shall be grateful if you can add our webpage URL into your lab website. As a token of appreciation, you will be rewarded by 1,000 EZcoupon™ points. For more information, please contact us by e-mail at web@genscript.com
