# Supplementary material for: Specific, sensitive and quantitative protein detection by in-gel fluorescence
Source: Nat Commun. 2023 May 2;14:2505. doi: 10.1038/s41467-023-38147-8 (PMC10154401; doi:10.1038/s41467-023-38147-8)
Supplement: Supplementary file 6 — Source Data [file 41467_2023_38147_MOESM6_ESM.zip › Source Data/Reagent analysis/U2441FG300-3-HPLC.pdf]

Sample Name :peptide Cy5.5
Sample ID :U2441FG300-3
Time Processed :10:18:50 AM
Month-Day-Year Processed :10/08/2020

Pump A : 0.065% trifluoroacetic in 100% water (v/v)
Pump B : 0.05% trifluoroacetic in 100% acetonitrile (v/v)
Total Flow:1 ml/min
Wavelength:220 nm

| Time | Module | Command | Value |
|---|---|---|---|
| 0.01 | Pumps | Pump A B.Conc | 35 |
| 25.00 | Pumps | Pump A B.Conc | 95 |
| 25.01 | Pumps | Pump A B.Conc | 95 |
| 31.00 | Pumps | Pump A B.Conc | 95 |
| 31.01 | Pumps | Pump A B.Conc | 35 |
| 40.00 | Pumps | Pump A B.Conc | 35 |
| 40.01 | Controller | Stop | |

<<Column Performance>>
<Detector A>
Column : Inertsil ODS-3 4.6 x 250 mm

Chromatogram

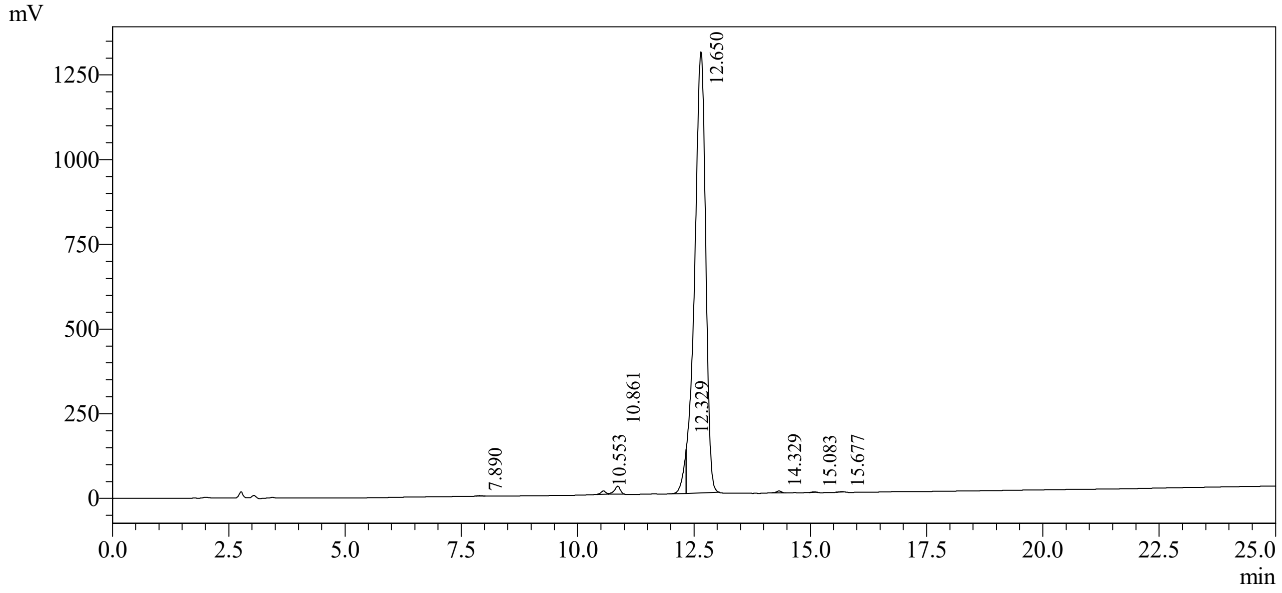


1 Detector A Channel 1 / 220nm

Peak Table

Detector A Channel 1 220nm

| Peak# | Ret. Time | Area | Height | Area % |
|---|---|---|---|---|
| 1 | 7.890 | 8708 | 1378 | 0.039 |
| 2 | 10.553 | 71241 | 9636 | 0.319 |
| 3 | 10.861 | 204175 | 23488 | 0.915 |
| 4 | 12.329 | 628195 | 119401 | 2.815 |
| 5 | 12.650 | 21348504 | 1302186 | 95.649 |
| 6 | 14.329 | 39626 | 5476 | 0.178 |
| 7 | 15.083 | 10575 | 1881 | 0.047 |
| 8 | 15.677 | 8703 | 1351 | 0.039 |
| Total | | 22319726 | 1464798 | 100.000 |
