# Supplementary material for: Specific, sensitive and quantitative protein detection by in-gel fluorescence
Source: Nat Commun. 2023 May 2;14:2505. doi: 10.1038/s41467-023-38147-8 (PMC10154401; doi:10.1038/s41467-023-38147-8)
Supplement: Supplementary file 6 — Source Data [file 41467_2023_38147_MOESM6_ESM.zip › Source Data/Reagent analysis/U2441FG300-3-MS.pdf]

Mass Spectrum

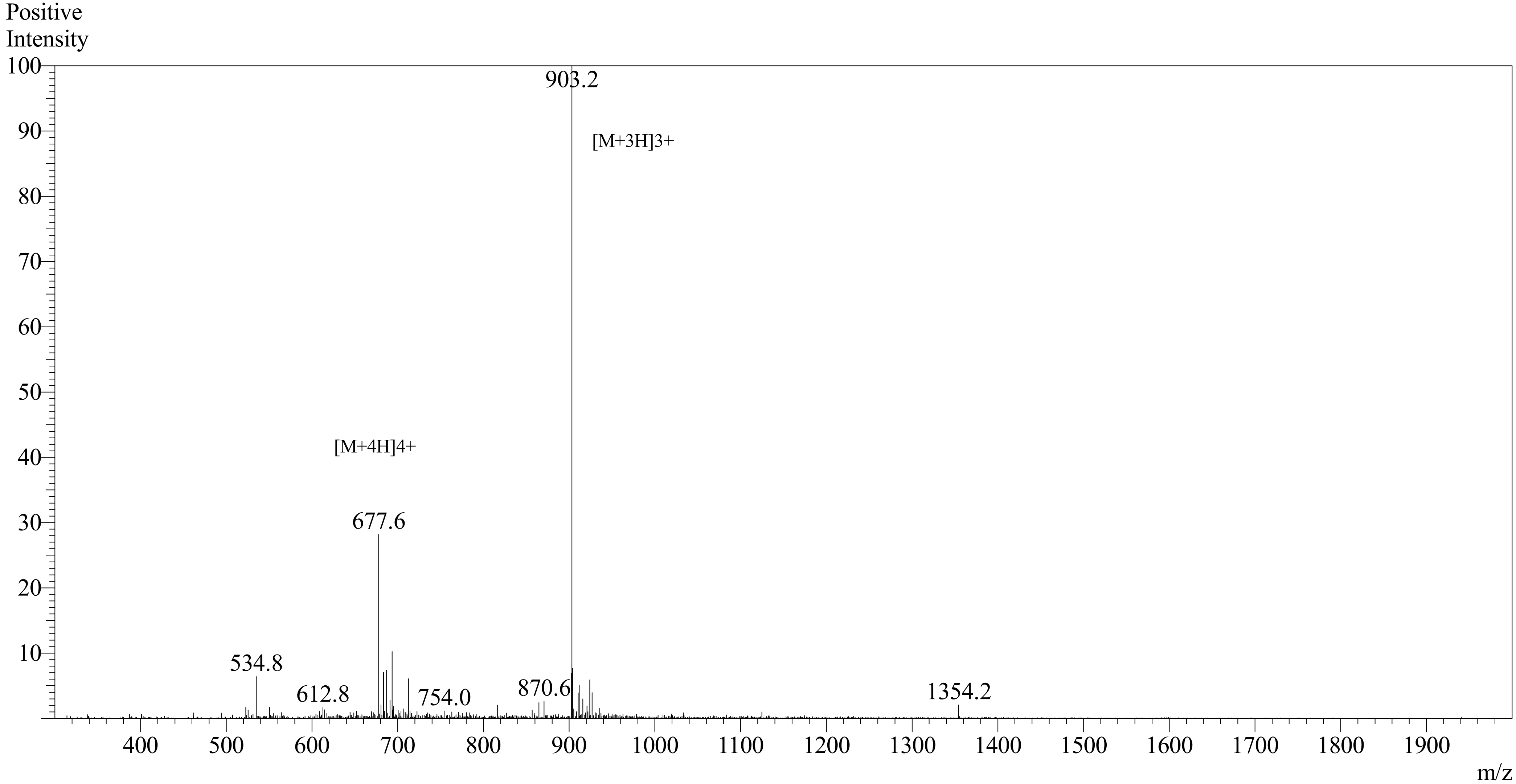

Sample Information	
Month-Day Processed :	09/29/20
Time Processed :	10:58:27 PM
Injection Volume :	0.3
Sample Name :	peptide Cy5.5
Sample ID :	U2441FG300-3
Theoretical MW :	2707.16
Observed MW :	2706.6

Interface	:ESI
Nebulizing Gas Flow	:1.5L/min
CDL Temp	:250
Block Temp	:200

Equipment	: GK11010007
Interface Bias	: +4.5 kV
Drying Gas Flow	:5 L/min
T.Flow	:0.2 ml/min
B.conc	:50%H2O/50%MeOH
