# Supplementary material for: Specific, sensitive and quantitative protein detection by in-gel fluorescence
Source: Nat Commun. 2023 May 2;14:2505. doi: 10.1038/s41467-023-38147-8 (PMC10154401; doi:10.1038/s41467-023-38147-8)
Supplement: Supplementary file 6 — Source Data [file 41467_2023_38147_MOESM6_ESM.zip › Source Data/Reagent analysis/U2441FG300-3-Peptide Content Report.pdf]

# Peptide Content Report

Sample Name: peptide Cy5.5
Sample ID: U2441FG300-3
Month-Day-Year Processed: 10-14-2020
Instrument: Vario MICRO Organic Elemental Analyzer

Result of detecting

| Times | N/% |
|---|---|
| Avg. | 11.55 |

Calculation Method:

The net peptide content/ % =N/%*MW(peptide)/(n*MW(nitrogen))

N/%: Nitrogencontentdetermined by elemental analysis;
MW(peptide): Theoretical Molecular Weight;
n: The number ofnitrogenelement;
MW (nitrogen): atomicweight (nitrogen);

Result of analyzing

| Times | N/% | The net peptide content/ % |
|---|---|---|
| Avg. | 11.55 | (11.55*2707.16)/(26*14)=85.90 |
| | | |
| Avg. | The net peptide content/ %=85.90 | |



GenScript USA, Inc.
860 Centennial Ave. Piscataway, NJ 08854
Tel: 1-732-885-9188
